# Urate‐Related Genetic Risk Modulates the Anti‐Aging Effects of Exogenous Nucleotides: Multi‐Omics Evidence From Older Adults

**DOI:** 10.1111/acel.70581

**Published:** 2026-06-09

**Authors:** Ruisheng Fu, Shuyue Wang, Yuxiao Wu, Xueying Qin, Tao Huang, Yong Li, Meihong Xu

**Affiliations:** ^1^ Department of Nutrition and Food Hygiene, School of Public Health Peking University Beijing China; ^2^ Department of Epidemiology and Biostatistics, School of Public Health Peking University Health Science Center Beijing China; ^3^ Key Laboratory of Epidemiology of Major Diseases (Peking University) Ministry of Education Beijing China; ^4^ Beijing Key Laboratory of Toxicological Research and Risk Assessment for Food Safety Peking University Beijing China

**Keywords:** DNA methylation age, exogenous nucleotides, multi‐omics, polygenic risk score, telomere length

## Abstract

Nucleotides—essential substrates for DNA repair, energy metabolism, and redox regulation—are emerging as nutritional modulators of biological aging. Yet, individual responses to exogenous nucleotide (NTs) supplementation remain poorly understood. Given that urate metabolism governs systemic oxidative balance, this study investigated whether genetic variability in urate regulation modifies the anti‐aging effects of NTs. In this secondary analysis of the TALENTs randomized controlled trial (121 adults aged 60–70 years; 19‐week intervention; NCT05243108), participants were stratified by urate polygenic risk score (UA‐PRS) to evaluate genotype‐dependent responses in DNA methylation age (DNAmAge) and leukocyte telomere length (LTL). A significant UA‐PRS × intervention interaction was observed for ΔDNAmAge (*p* = 0.0114) and ΔLTL (*p* = 0.0271). NTs supplementation reduced DNAmAge in the High‐PRS group (*β* = −5.10, *p* = 0.00013) and preserved telomere length in the Low‐PRS group (*β* = 0.31, *p* = 0.0043). Multi‐omics analyzes revealed that NTs modulated glucose‐transport and pentose phosphate pathways in High‐PRS individuals, while enhancing immune regulation and reducing GDF15 and IL‐1β levels in Low‐PRS individuals. These findings suggest that genetic variation in urate metabolism determines whether NTs are preferentially utilized for redox and epigenetic maintenance or for immune and inflammatory regulation, supporting a precision‐nutrition model for delaying biological aging.

## Graphical abstract

In the TALENTs trial, 19‐week exogenous nucleotide supplementation produced UA‐PRS‐dependent anti‐aging responses: reduced DNA methylation age in High‐PRS individuals and preserved telomere length with improved immune–inflammatory profiles in Low‐PRS individuals, supporting precision nutrition for healthy aging.

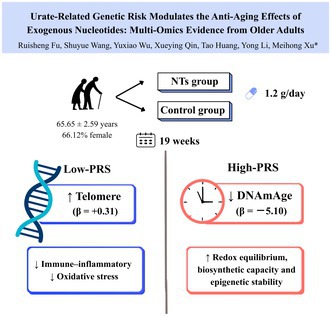

## 1 Introduction

Aging is a multidimensional biological process, and with the accelerating global aging population, effective interventions to delay aging have become an urgent public health priority (O'Caoimh et al. 2021). Aging involves not only physiological decline but also alterations in key biological hallmarks such as genomic instability, mitochondrial dysfunction, and dysregulated nutrient sensing (Kroemer et al. 2025).

Among various biomarkers of aging, DNA methylation age (DNAmAge) and leukocyte telomere length (LTL) are widely recognized as core molecular indicators for evaluating biological aging (Moqri et al. 2023). DNAmAge reflects epigenetic alterations associated with aging and has been closely linked to energy metabolism and metabolic syndrome (Horvath and Raj 2018; Duan et al. 2022), whereas LTL represents cumulative cellular replicative and stress‐induced damage and shows negative associations with chronic inflammation (Daios et al. 2022; Fasching 2018). Both biomarkers have been extensively applied in aging surveillance and intervention research, providing quantitative measures for assessing the anti‐aging effects of nutritional and lifestyle interventions (Loughlin et al. 2025). Therefore, identifying nutritional strategies that can modulate DNAmAge and LTL holds great potential for delaying biological aging.

Nucleotides, as essential components of DNA and RNA, play critical roles in cellular repair, energy metabolism, and genomic stability (Ding et al. 2021). With advancing age, endogenous synthesis and absorption efficiency of nucleotides decline, potentially leading to functional insufficiency and impaired cellular processes (Sánchez‐Pozo and Gil 2002; Yamamoto et al. 1997). Preclinical studies have demonstrated that exogenous nucleotides (NTs) can enhance telomerase activity, delay telomere shortening, and improve cellular repair and metabolic homeostasis (Xu, Liang, et al. 2013; Bryan 2023). NTs have also been shown to elevate intracellular NAD^+^ levels and activate the NAD^+^/SIRT1/PGC‐1α signaling pathway, thereby improving mitochondrial function and reducing oxidative stress (Frankic et al. 2006; Qu et al. 2025).

Extending these findings, recent randomized controlled trials have provided clinical evidence supporting the anti‐aging potential of NTs. NTs supplementation significantly reduced DNAmAge and improved insulin sensitivity (Wang et al. 2025). However, findings regarding LTL remain inconsistent, possibly due to differences in intervention duration, individual metabolic status, and genetic background. Such heterogeneity underscores the importance of considering genetic predisposition in developing precision nutrition strategies to optimize anti‐aging interventions.

In recent years, growing attention has been directed toward the interaction between genetics and dietary interventions, with accumulating evidence indicating that single‐nucleotide polymorphisms (SNPs) can modulate individual responses to diet (Duarte et al. 2025). To capture overall genetic susceptibility, polygenic risk scores (PRS) have been established as composite measures integrating multiple SNP effects (Janssens 2019); for instance, PRS derived from obesity‐associated loci have been reported to influence metabolic responses to dietary interventions (Seral‐Cortes et al. 2020; Sekar et al. 2024; Franck et al. 2019). Genetic variations in the APOA1 gene can influence high‐density lipoprotein (HDL) levels, and different genotypes lead to individual differences in response to long‐chain omega‐3 fatty acid intake (Xu et al. 2016; Wilson et al. 1996; Lumsden et al. 2020). These findings suggest that genetic susceptibility may underlie inter‐individual variability in the anti‐aging effects of NTs supplementation, underscoring the importance of investigating gene–nutrient interactions in biological aging.

Exogenous nucleotides (NTs) participate in purine metabolism, in which serum urate (UA) serves as the terminal degradation product in humans (Maiuolo et al. 2016). Epidemiological evidence from large cohorts has revealed a U‐shaped association between serum UA levels and aging‐related outcomes, including mortality and biological aging indices (Hu et al. 2020). This duality reflects the oxidant–antioxidant paradox of UA, functioning as an extracellular antioxidant but acting as a pro‐oxidant under certain intracellular conditions (Xu et al. 2025).

Given the polygenic nature of UA regulation, UA‐based polygenic risk scores (UA‐PRS) have been developed to capture genetic variability in urate metabolism (Tin et al. 2019). In this study, a UA‐PRS was adopted to characterize individual differences in nucleotide metabolic capacity and to examine whether this genetic background influences the anti‐aging effects of NTs supplementation. Previous findings linking UA levels to cognitive aging and longevity further highlight its complex role in biological aging (Chen et al. 2021).

Accordingly, this study aimed to evaluate the effects of exogenous nucleotide supplementation on biological aging, assessed by DNA methylation age and leukocyte telomere length, and to determine whether these effects vary according to genetic susceptibility defined by UA‐PRS. This investigation provides an integrated framework for understanding gene–nutrient interactions in the modulation of aging trajectories.

## 2 Methods

### 2.1 Study Design and Participants

This study represents a secondary analysis based on the TALENTs randomized controlled trial, which was conducted among community‐dwelling older adults in Chengdu, Sichuan Province, China. The trial design and methodological details have been described elsewhere (Wang et al. 2025).

Participants were men and women aged 60–70 years who were generally healthy and free from severe physical or mental disorders. Eligibility criteria included no prior consumption of nucleotide‐containing supplements or similar health products, the ability to adhere to study procedures, and the provision of written informed consent. Individuals were excluded if they had any major clinical condition—such as autoimmune, cardiovascular, or cerebrovascular disease, hepatic or renal dysfunction, or malignancy—or if they had substantial visual or hearing impairment that could hinder communication. Additional exclusion criteria comprised participation in another clinical study within the preceding six months or the use of medications or food products that might influence study outcomes.

All participants signed written informed consent before enrollment and retained the right to withdraw at any time. The study protocol was reviewed and approved by the Biomedical Ethics Committee of Peking University (approval number: IRB00001052‐21114) and was conducted in accordance with the principles of the Declaration of Helsinki. The trial was registered at ClinicalTrials.gov (identifier: NCT05243108).

### 2.2 Intervention

The intervention and placebo capsules were manufactured by the Hainan Double‐D Geneo Life Science Research Center. Both preparations were identical in appearance, taste, and packaging, differing only in the presence or absence of nucleotides in the active capsules. Each capsule contained 0.4 g of material, and participants were instructed to take four capsules per day. The placebo consisted solely of 0.4 g of excipients, whereas the active formulation contained 0.3 g of nucleotides combined with 0.1 g of excipients.

The nucleotide mixture was composed of 5′‐AMP, 5′‐CMP, 5′‐GMPNa_2_, and 5′‐UMPNa_2_ in a ratio of 16:41:19:24, reflecting the natural nucleotide distribution found in human breast milk. The formulation met national regulatory standards for medical foods and infant formula (Xu, Zhao, et al. 2013; Tressler et al. 2003). The total daily dose of 1.2 g fell within the range approved for health food products. The supplementation period lasted 19 weeks, and only participants who achieved a compliance rate of ≥ 95% were included in the final analyzes.

### 2.3 Measurements

All measurement methods are summarized in Table S1 and described in detail below.

#### 2.3.1 Dietary Assessment

Dietary intake was evaluated at both baseline and post‐intervention using a semi‐quantitative food frequency questionnaire (FFQ) that captured habitual intake over the preceding year. To enhance accuracy, participants also completed three consecutive 24‐h dietary recalls at each assessment point for cross‐validation of reported consumption. Special attention was paid to nucleotide‐rich foods such as meat, fish, and legumes (Fan et al. 2024). Because no universally standardized method exists for quantifying dietary nucleotide intake, estimation was performed according to the procedure outlined by Ding et al. (Ding et al. 2021), in which the nucleotide content was approximated based on total protein intake after subtracting other nitrogenous constituents, including amino acids and B vitamins.

#### 2.3.2 Assessment of Biological Aging Biomarkers

To evaluate biological aging, two molecular biomarkers were assessed: DNA methylation age (DNAmAge) and leukocyte telomere length (LTL).

##### 2.3.2.1 DNA Methylation Profiling

Whole‐genome bisulfite sequencing (WGBS) was performed on fasting venous blood samples to quantify genome‐wide methylation levels. DNA was extracted from clotted blood and bisulfite converted using the EZ DNA Methylation‐Gold Kit (Zymo Research, USA). Sequencing libraries were prepared following the manufacturer's instructions and subjected to quality control prior to high‐throughput sequencing. Raw reads were filtered with SOAPnuke to remove low‐quality sequences, and the remaining clean reads were aligned to the human reference genome using Bismark. Methylation ratios at each CpG site were calculated, and coverage maps were generated for downstream analysis.

Four principal component (PC)‐corrected epigenetic clocks—Horvath (Horvath 2013), Hannum (Hannum et al. 2013), GrimAge (Lu et al. 2019), and DNAm PhenoAge (Levine et al. 2018)—were computed using the R package dnaMethyAge (https://github.com/yiluyucheng/dnaMethyAge) (Lehne et al. 2016). For each participant, the median value of the four clocks (Median DNAmAge) was used as the composite index of epigenetic age. This approach minimizes biases inherent to individual clocks and improves precision in estimating biological aging.

##### 2.3.2.2 Telomere Length Measurement

Relative leukocyte telomere length (LTL) was determined by quantitative PCR (qPCR) using assay kits from Shanghai Yihe Biotechnology Co. Ltd. The telomere (T) to single‐copy gene (S) ratio (T/S) was calculated for each sample to indicate relative telomere length, reflecting cellular senescence and replicative capacity.

#### 2.3.3 Serum Urate Measurement

Serum urate (UA) concentration was determined using the uricase–peroxidase enzymatic colorimetric method with commercial diagnostic kits (Fosun Diagnostics, China) on an AU5800 automatic biochemical analyzer (Beckman Coulter, USA). All measurements were performed in duplicate, and results were expressed in μmol/L.

#### 2.3.4 Immune Marker Assessments

##### 2.3.4.1 T Lymphocyte Subset

Cell‐mediated immunity was evaluated through quantification of CD3^+^, CD4^+^, and CD8^+^ T lymphocyte subsets by flow cytometry using a Bio‐Rad ZE5 flow cytometer (Bio‐Rad Laboratories, USA) and fluorescence‐labeled BD Pharmingen antibodies. Forward and side scatter parameters were applied for gating, and results were expressed as the percentage of total lymphocytes.

##### 2.3.4.2 Inflammatory and Humoral Immune Marker

Serum concentrations of IL‐1β, TNF‐α, and GDF‐15 were measured using a Quantibody Custom Array (QAH‐CUST) platform (RayBiotech, USA) according to the manufacturer's protocol. Fluorescence signals were detected with a GenePix 4200A microarray scanner, and cytokine levels were calculated from standard curves. Immunoglobulin G (IgG) was determined via immunoturbidimetry on a Hitachi 7180 biochemical analyzer (Hitachi High‐Tech, Japan), where antigen–antibody complex formation produces turbidity proportional to immunoglobulin concentration.

#### 2.3.5 Transcriptomic Analysis

Fasting venous blood samples were collected for transcriptomic profiling. Total RNA was isolated and immediately stored at −80°C, followed by cold‐chain transport to BGI (Shenzhen, China) for high‐throughput RNA sequencing to examine gene expression alterations related to aging. Raw sequencing reads underwent quality control (QC) using SOAPnuke, which filtered out reads containing adapter sequences, those with more than 5% unidentified bases (N), and reads with over 20% of bases having a Phred quality score below 15. Clean, high‐quality reads were subsequently mapped to the reference genome using HISAT (Hierarchical Indexing for Spliced Alignment of Transcripts). HISAT utilizes a Burrows–Wheeler transform combined with a Ferragina–Manzini (FM) index strategy, integrating both global and local indexing to ensure highly efficient and accurate alignment. The algorithm first performs a coarse mapping with the global index, followed by refined local alignment to enhance sensitivity and completeness of transcript reconstruction.

#### 2.3.6 Metabolomics Analysis

Fasting venous blood samples were collected, and serum was isolated using separation gel tubes. The samples were aliquoted, stored at −80°C, and transported under a cold chain to Biotree Biotechnology (Shanghai, China) for metabolomic profiling, focusing on small‐molecule metabolites related to nucleotide metabolism.

Targeted metabolomics was performed using LC–MS/MS on a 600 MRM platform (Biotree, Shanghai, China). After thawing on ice, serum samples were vortex‐mixed and extracted with an acetonitrile–methanol solution containing isotope‐labeled internal standards. The extracts were processed through ultrasonication, incubation, centrifugation, and vacuum drying. Residues were then reconstituted in 60% acetonitrile, centrifuged again, and the resulting supernatants were analyzed by LC–MS/MS.

Chromatographic separation was achieved on an ACQUITY UPLC H‐Class system (Waters, USA) equipped with a Waters Atlantis Premier BEH Z‐HILIC column, using mobile phases containing ammonium acetate (pH 9). Mass spectrometric detection was conducted on a SCIEX 6500 QTRAP+ triple quadrupole equipped with an IonDrive Turbo V ESI source operating in multiple reaction monitoring (MRM) mode. Data acquisition and quantification were performed using SCIEX Analyst WorkStation Software (v1.7.2) and BIOTREE Bio Bud (v2.1.4). Metabolite concentrations were calculated based on the final measured concentration, dilution factor, concentration factor, final volume, and sample weight, and expressed as nmol/g.

### 2.4 Genotyping and Calculating the Fasting Blood Glucose Polygenic Risk Score

Genomic DNA was extracted from peripheral blood samples using the TIANGEN DNA extraction kit following standard protocols, and DNA quality was assessed by spectrophotometry (OD260/280 ratio 1.7–2.0), Qubit quantification, and agarose gel electrophoresis to ensure integrity. Qualified samples were genotyped for genome‐wide single nucleotide polymorphisms (SNPs) using a high‐throughput Illumina genotyping array.

Quality control of the genotyping arrays indicated reliable experimental performance. Staining and extension controls showed normal sensitivity and efficiency, target removal controls confirmed effective template separation, and hybridization controls demonstrated expected signal gradients across concentrations. Together, these results verified the overall accuracy and reliability of the genotyping data, ensuring suitability for downstream PRS calculation.

The process included DNA amplification, fragmentation, hybridization to probe arrays, and fluorescence‐based scanning with the Illumina iScan system to generate SNP data, which were subsequently used to construct PRS. PRS for serum urate were calculated based on single nucleotide polymorphisms (SNPs) previously identified in East Asian populations (Tin et al. 2019). A total of 46 SNPs associated with serum urate were reported, of which 36 were available in our dataset. For each SNP, the corresponding effect size (*β*) derived from genome‐wide association studies (GWAS) was extracted. Individual PRS values were computed using PLINK 1.9 with the ‐‐score function, according to the following equation (Purcell et al. 2007):

$${\mathrm{PRS}}_{i}=\sum_{j=1}^{n}\left(\beta_{j}\cdot G{\text{enotype}}_{\mathrm{ij}}\right)$$

where $\beta_{j}$ denotes the GWAS effect size of the j‐th SNP, and $G{\text{enotype}}_{\mathrm{ij}}$ represents the genotype score (0, 1, or 2) of individual i at the corresponding locus. The resulting continuous PRS provides an estimate of genetic predisposition to serum urate.

### 2.5 Statistical Analyzes

Continuous variables are presented as mean ± standard deviation (SD), and categorical variables as frequencies with percentages. The Shapiro–Wilk test was used to assess normality. Between‐group comparisons were conducted using the *χ* ^2^ test, Fisher's exact test, or independent‐samples *t*‐test, as appropriate. No data transformation or outlier exclusion was applied.

To validate the UA‐PRS, its association with serum urate (UA) concentration was examined using multivariable linear regression, adjusting for age, sex, and dietary nucleotide intake. To evaluate whether genetic background modified the effects of exogenous nucleotide (NTs) supplementation, interaction terms (UA‐PRS × intervention) were included in linear regression models, further adjusting for age, sex, and dietary nucleotide intake.

Participants were stratified into High‐ and Low‐PRS groups based on the median score. Within each stratum, changes in biological‐aging markers (ΔDNAmAge and Δtelomere length) were analyzed using generalized estimating equations (GEE) with an exchangeable correlation structure, controlling for age, sex, and dietary nucleotide intake.

For the transcriptomic analysis, differential gene expression was assessed using DESeq2, with thresholds set at |log2 fold change| > 1 and adjusted *p* value (*Q* value) < 0.05. The DESeq2 algorithm models count data within a negative binomial distribution framework, following the approach described by Love et al. (Love et al. 2014). Identified differentially expressed genes (DEGs) were subsequently annotated and subjected to pathway enrichment analysis. Functional annotation was performed using the Gene Ontology (GO) database to determine significantly enriched biological processes.

For immune‐related indicators, GEE models were applied to evaluate NTs‐induced changes within each PRS stratum, adjusting for age, sex, and dietary nucleotide intake. To further explore the relationship between immune alterations and biological aging markers, multivariable linear regression analyzes were performed to assess associations between changes in immune parameters and changes in telomere length, controlling for age and sex.

For metabolomics, intervention‐related changes within each PRS stratum were assessed using GEE models adjusted for age, sex, and dietary nucleotide intake. Differential metabolites (*p* < 0.05) were subjected to pathway enrichment analysis using MetaboAnalyst (v6.0) based on the Small Molecule Pathway Database (SMPDB; http://smpdb.ca/) (Jewison et al. 2014).

All statistical analyzes were performed using R (version 4.4.0), and a two‐sided *p* ≤ 0.05 was considered statistically significant.

## 3 Results

### 3.1 Baseline Characteristics and Validation of UA‐PRS

A total of 121 participants were enrolled in the study, including 62 in the control group and 59 in the NTs intervention group. Baseline characteristics and changes in outcome measures over the follow‐up period are presented in Table 1. The mean age of the study population was approximately 65 years, with women accounting for about 66%. There were no significant differences between the two groups in terms of age, sex distribution (*p* > 0.05).

**TABLE 1:** Baseline characteristics and follow‐up changes of participants in the TALENTs study.

Variable	Time	Overall		Control group		NTs group		*p*
		*N*	Mean ± SD	*N*	Mean ± SD	*N*	Mean ± SD	
Age (years)	—	121	65.65 ± 2.59	62	65.74 ± 2.58	59	65.55 ± 2.63	0.685
Female, *n* (%)	—	121	80 (66.12)	62	41 (66.13)	59	39 (66.10)	0.997
UA‐PRS	—	121	0.13 ± 0.3	62	0.1 ± 0.32	59	0.16 ± 0.27	0.314
Median DNAmAge (years)	T0	121	58.52 ± 4.86	62	57.26 ± 5.16	59	59.85 ± 4.18	**0.0029**
	T1	83	59.04 ± 6.41	42	58.62 ± 6.1	41	59.47 ± 6.77	0.552
	T2	118	54.9 ± 5.48	61	55.14 ± 4.83	57	54.64 ± 6.14	0.622
	T2‐ T0	118	−3.72 ± 5.74	61	−2.21 ± 5.34	57	−5.33 ± 5.76	**0.0029**
Leukocyte Telomere length (T/S ratio)	T0	121	3.26 ± 0.5	62	3.27 ± 0.55	59	3.25 ± 0.45	0.789
	T1	118	3.04 ± 0.46	61	3.01 ± 0.42	57	3.08 ± 0.49	0.432
	T2	118	2.93 ± 0.36	61	2.88 ± 0.35	57	2.97 ± 0.36	0.174
	T2‐ T0	118	−0.32 ± 0.48	61	−0.38 ± 0.55	57	−0.27 ± 0.4	0.208
UA (μmol/L)	T0	121	289.94 ± 63.94	62	284.58 ± 57.3	59	295.58 ± 70.3	0.349
	T1	118	296.35 ± 72.91	61	276.59 ± 70.22	57	317.49 ± 70.3	**0.002**
	T2	118	309.83 ± 68.77	61	298.52 ± 63.22	57	321.93 ± 72.86	0.0658
Nucleotide intake (mg)	T0	121	1122.09 ± 1694.54	62	1054.17 ± 1793.71	59	1193.46 ± 1595.94	0.652
	T2	118	1624.7 ± 2150.52	61	1731.06 ± 1994.25	57	1512.94 ± 2315.35	0.581

*Note:* T0 refers to the baseline measurement, T1 represents the midpoint measurement, T2 represents the endpoint measurement. T2‐T0 represents the change from T0 to T2. The *p*‐values are derived from *t*‐tests that compare the changes between NTs group and Control group. Bolded entries fall below the significance threshold of *p* < 0.05. The calculation method for nucleotides is as described in the literature (Ding et al. 2021).

Abbreviations: UA‐PRS, the serum urate polygenic risk score; UA, serum urate.

The UA‐PRS used in this study was constructed based on genome‐wide association study (GWAS) results of UA in East Asian populations. The mean UA‐PRS for the overall study population was 0.13 ± 0.3. No significant difference in UA‐PRS was observed between the intervention and control groups.

At baseline, there were no significant between‐group differences in LTL, UA, or dietary nucleotide intake (*p* > 0.05). The intervention and control groups were comparable in demographic and metabolic characteristics, ensuring a balanced starting point for subsequent analyzes.

To assess the applicability of the UA‐PRS in this study population, multivariable linear regression adjusted for age, sex, and dietary nucleotide intake showed that UA‐PRS was significantly and positively associated with UA (*β* = 53.68, 95% CI: 21.88 to 85.47, *p* = 0.00111), indicating good predictive performance of the score in this cohort (Table S2).

### 3.2 Interaction Between UA‐PRS and NTs Supplementation in Modulating Epigenetic and Telomeric Aging Markers

To assess whether genetic background modified the intervention effect, UA‐PRS was first analyzed as a continuous variable. A significant interaction was observed with ΔMedian DNAmAge (*p* = 0.0114; Table S3), indicating that the effect of the intervention on aging varied by genetic risk. Similarly, a significant interaction was also detected for Δtelomere length (*p* = 0.0271; Table S4), suggesting that the influence of genetic background extended to telomere‐related aging dynamics as well.

**FIGURE 1:**
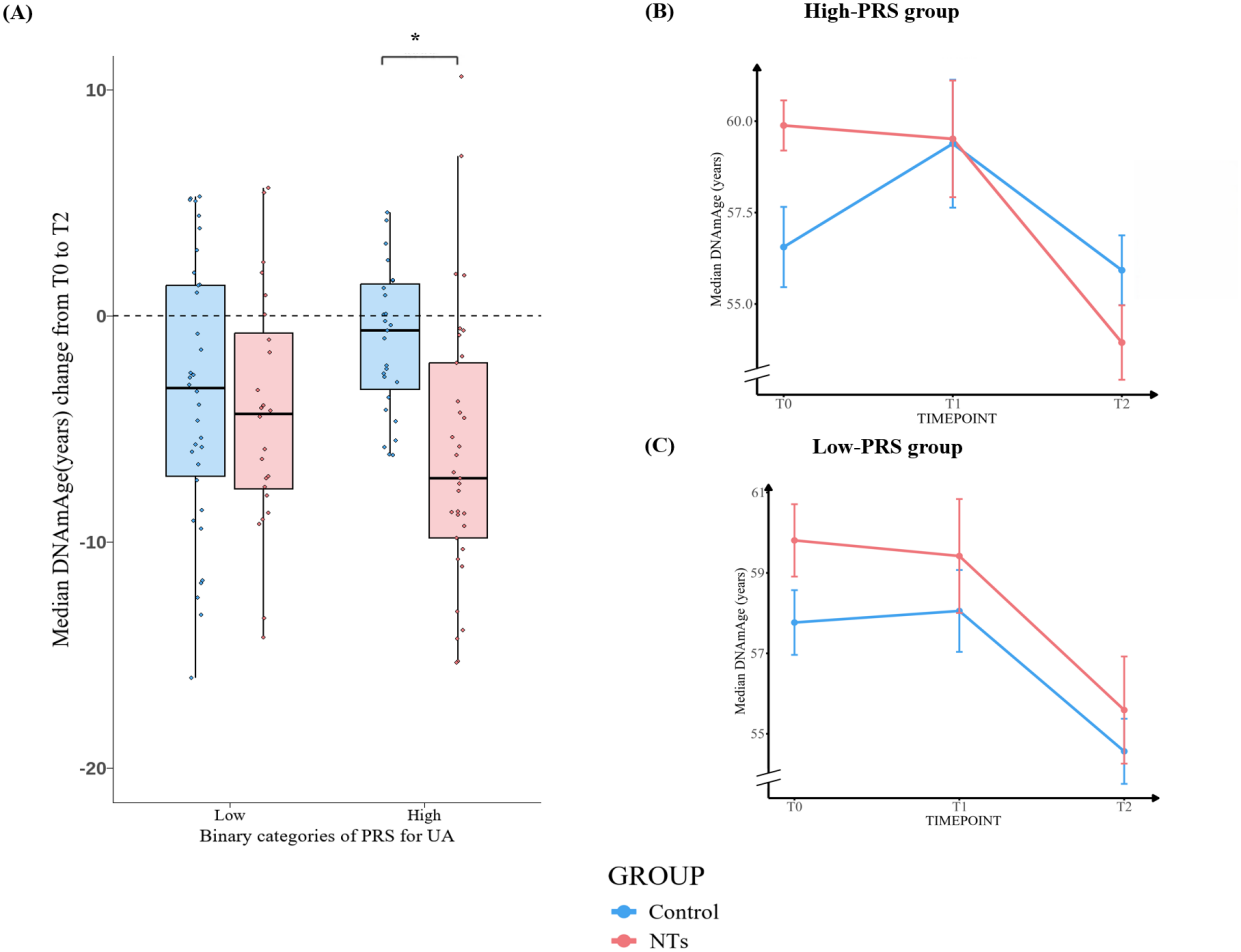
Impact of NTs intervention on Median DNAmAge stratified by UA‐PRS group. (A) Changes in Median DNAmAge (T2–T0) in the Control and NTs groups, stratified by binary categories of UA‐PRS. (B) Longitudinal changes in Median DNAmAge at T0, T1, and T2 in the High‐PRS group (*n* = 60). (C) Longitudinal changes in Median DNAmAge at T0, T1, and T2 in the Low‐PRS group (*n* = 61). * T0 refers to the baseline measurement, T1 represents the midpoint, and T2 indicates the endpoint of the intervention.

Participants were then stratified into High‐PRS and Low‐PRS groups by the median score. In the High‐PRS group, NTs supplementation significantly reduced Median DNAmAge compared with control (*β* = −5.10, 95% CI −7.71 to −2.48, *p* = 0.00013; Figure 1A). Examined longitudinally, Median DNAmAge in the NTs group decreased from 59.88 ± 4.00 to 53.95 ± 5.84 (T2–T0: −6.11 ± 6.07, *p* = 0.00013), while it increased in the control group (T2–T0: −0.63 ± 4.11) (Figure 1B). In the Low‐PRS group, changes were minimal and not significant in either arm (*p* = 0.59; Figure 1C). Detailed longitudinal changes in DNAmAge and telomere length between intervention and control arms, along with the corresponding GEE model results, are presented in Tables S5 and S6.

**FIGURE 2:**
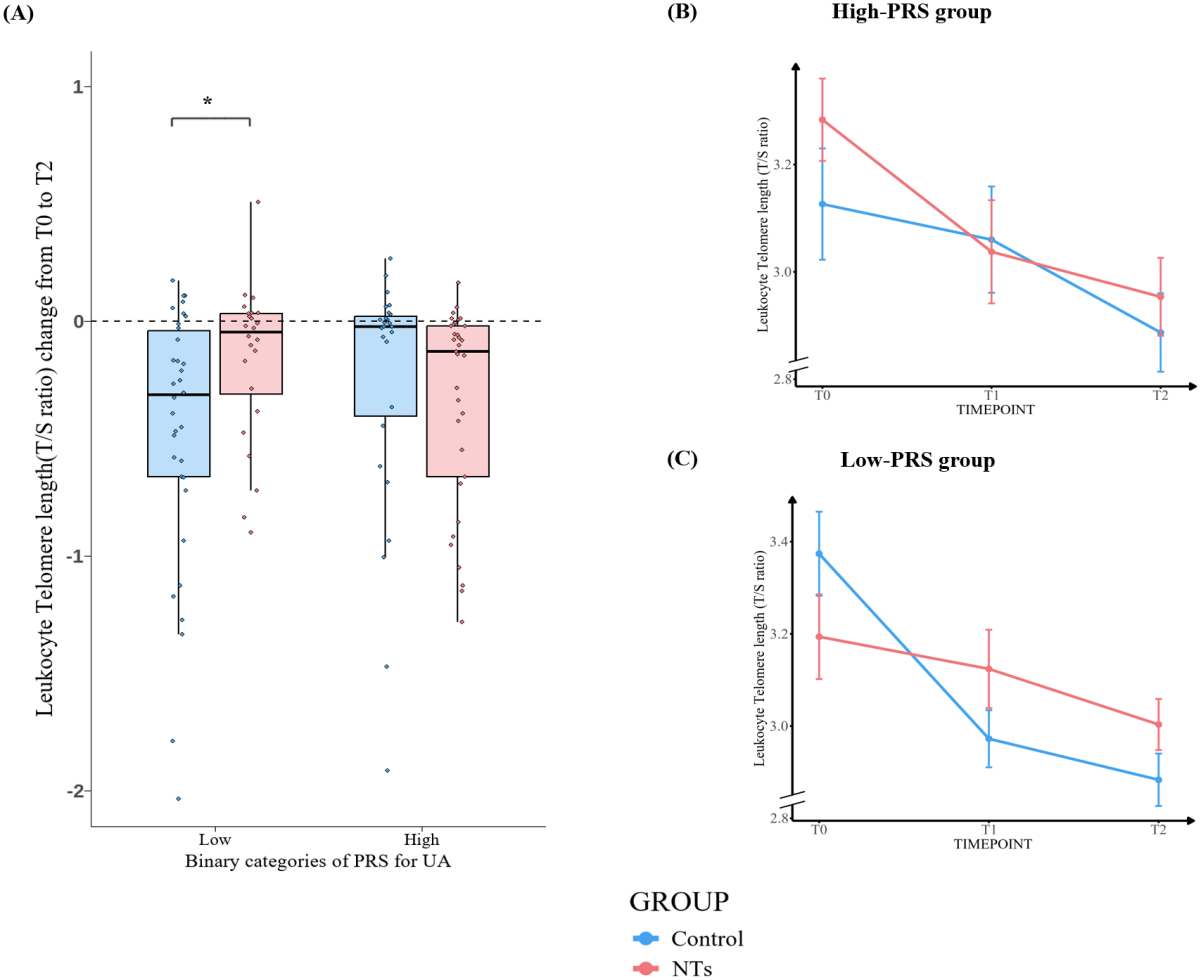
Impact of NTs intervention on telomere length stratified by UA‐PRS group. (A) Changes in telomere length (T2–T0) in the Control and NTs groups, stratified by binary categories of UA‐PRS. (B) Longitudinal changes in telomere length at T0, T1, and T2 in the High‐PRS group (*n* = 60). (C) Longitudinal changes in telomere length at T0, T1, and T2 in the Low‐PRS group (*n* = 61). * T0 refers to the baseline measurement, T1 represents the midpoint, and T2 indicates the endpoint of the intervention.

However, the results for telomere length showed a distinct pattern. In the Low‐PRS group, NTs supplementation significantly attenuated telomere shortening compared with the control (*β* = 0.31, 95% CI 0.10 to 0.53, *p* = 0.0043; Figure 2A). Examined longitudinally, telomere length in the NTs group declined slightly from 3.19 ± 0.46 to 3.00 ± 0.27 (T2–T0: −0.16 ± 0.33, *p* = 0.0074), whereas a greater reduction was observed in the control group (T2–T0: −0.48 ± 0.55) (Figure 2B). In the High‐PRS group, changes were minimal and not significant in either arm (*p* = 0.53; Figure 2C).

### 3.3 Differential Transcriptomic Responses to NTs Intervention by UA‐PRS Risk Group

To further explore why NTs supplementation exerted distinct anti‐aging effects between individuals with different UA‐PRS risk background, differentially expressed genes (DEGs) were analyzed separately in the High‐PRS and Low‐PRS groups. In the High‐PRS group, five differentially expressed transcript features were identified, corresponding to four unique genes, including one upregulated transcript and four down regulated transcripts (Table S7). Specifically, two SEC14L1 transcripts were downregulated (NM_001204410: log2FC = −1.10, *p*adj = 2.67 × 10^−6^; NM_001144001: log2FC = −1.10, *p*adj = 4.94 × 10^−5^), while PPP4R2 (NM_001318028: log2FC = −1.38, *p*_adj_ = 5.35 × 10^−6^) and ORC5 (NM_181747: log2FC = −1.19, *p*adj = 0.032) were also downregulated. In contrast, GPBP1 was upregulated (NM_001127236: log2FC = 1.57, *p*adj = 0.012). Given the limited number of differentially expressed features in this subgroup, subsequent interpretation was restricted to the gene level. These changes involved genes related to lipid‐associated intracellular transport, protein phosphatase‐associated regulation, cellular stress‐related processes, and DNA replication‐associated functions, suggesting that NTs supplementation may induce specific transcriptional responses in individuals with a High‐PRS background. Nevertheless, these findings should be regarded as exploratory and require further validation.

**FIGURE 3:**
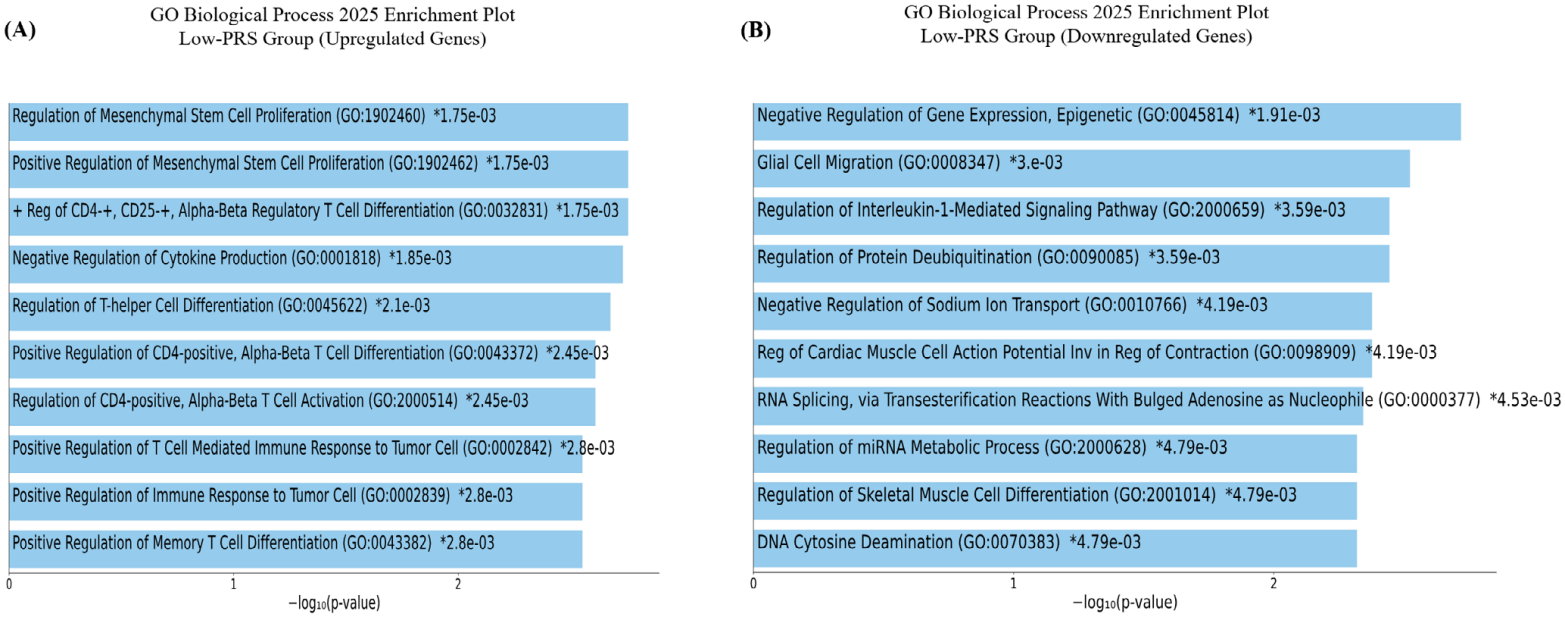
GO Biological Process 2025 enrichment analysis of differentially expressed genes following NTs intervention in Low‐PRS group. (A) Enrichment results of upregulated genes in the Low‐PRS group. (B) Enrichment results of downregulated genes in the Low‐PRS group.

In the Low‐PRS group, a total of 19 DEGs were identified, including 7 upregulated and 12 downregulated genes (Table S8). The upregulated DEGs were mainly enriched in Regulation of Mesenchymal Stem Cell Proliferation (GO:1902460), Positive Regulation of Mesenchymal Stem Cell Proliferation (GO:1902462), and Positive Regulation of CD4‐positive, CD25‐positive, Alpha‐Beta Regulatory T Cell Differentiation (GO:0032831), suggesting that NTs supplementation activated genes associated with stem‐cell proliferation and T‐cell differentiation (Figure 3A).

Meanwhile, the downregulated DEGs were significantly enriched in Negative Regulation of Gene Expression, Epigenetic (GO:0045814), Glial Cell Migration (GO:0008347), and Regulation of Interleukin‐1‐Mediated Signaling Pathway (GO:2000659), indicating that NTs supplementation reduced the expression of genes involved in epigenetic regulation, glial cell activity, and inflammatory signaling in the Low‐PRS group (Figure 3B).

### 3.4 NTs Supplementation Modulated Immune Homeostasis and Inflammatory Response Predominantly in the Low‐PRS Group

Given that NTs supplementation delayed telomere shortening and transcriptomic enrichment indicated immune‐ and inflammation‐related pathways in the Low‐PRS group, immune parameters were further examined to assess systemic immune modulation. In contrast, no significant immune alterations were observed in the High‐PRS group.

In the Low‐PRS group, NTs supplementation was associated with changes in adaptive immune markers. CD3^+^CD4^+^ T cells increased (*β* = 2.55, 95% CI 0.07–5.03, *p* = 0.044), and the CD4^+^/CD8^+^ ratio was elevated (*β* = 0.29, 95% CI 0.06–0.52, *p* = 0.014). Both indicators reflect T‐cell immune balance and adaptive immune status. IgG showed a mild but significant reduction (*β* = −0.66, 95% CI −1.32–0.00, *p* = 0.049), consistent with lower chronic immune activation.

**FIGURE 4:**
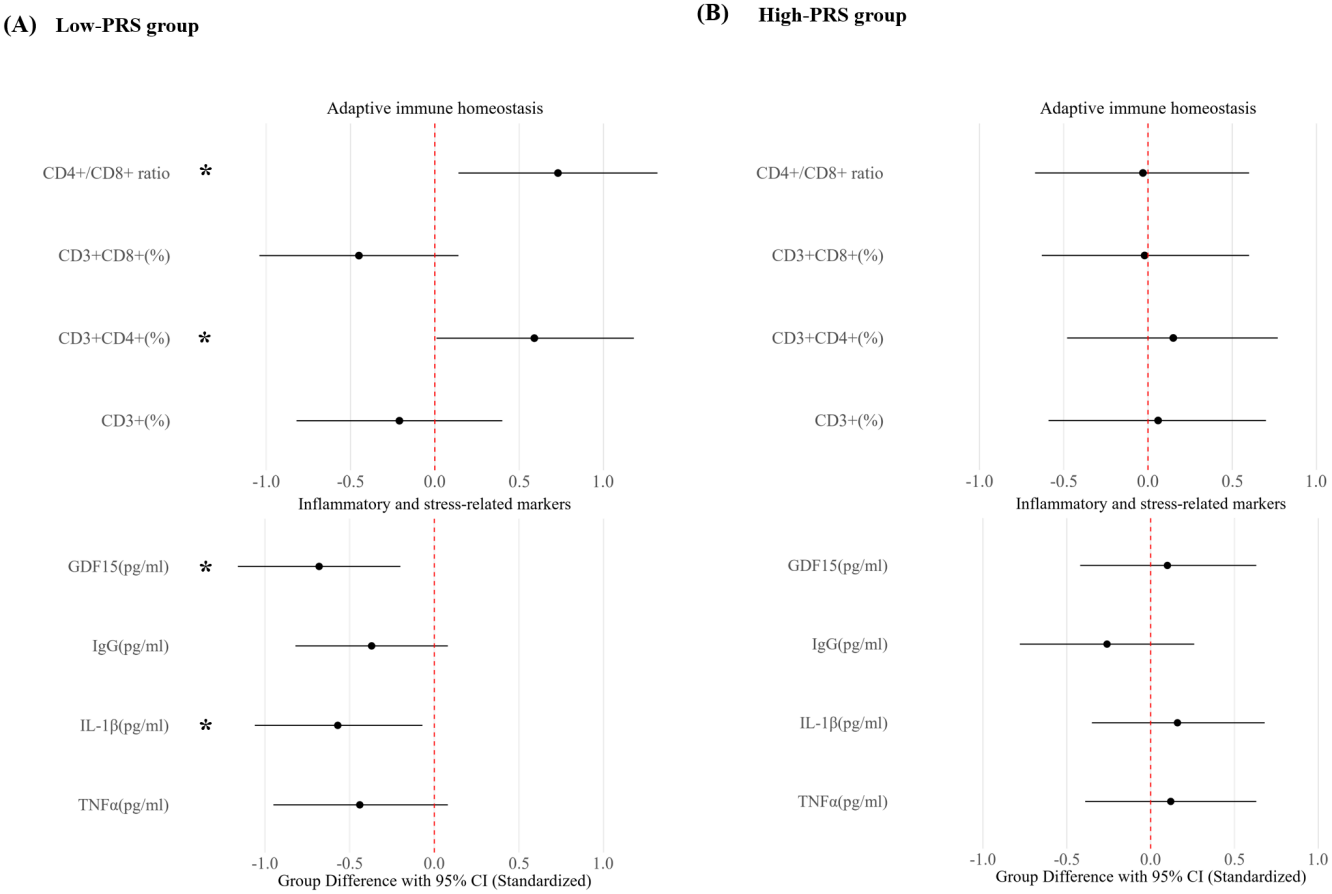
Effects of NTs supplementation on immune and inflammatory biomarkers stratified by UA‐PRS group. GEE model–estimated changes (*β*±95% CI) in T lymphocyte subsets and inflammatory or humoral immune markers are shown for the Low‐PRS (A) and High‐PRS (B) groups. * CD, cluster of differentiation; IL‐1β, interleukin‐1 beta; TNF‐α, tumor necrosis factor alpha; GDF‐15, growth differentiation factor 15; IgG, immunoglobulin.

NTs supplementation also resulted in reductions of circulating GDF15 (*β* = −105.72, 95% CI −179.01 to −32.42, *p* = 0.0047) and IL‐1β (*β* = −92.27, 95% CI −177.71 to −6.84, *p* = 0.034). These molecules are known markers of inflammatory and cellular stress responses. Their decrease indicates a shift toward a lower systemic inflammatory state. Taken together, NTs supplementation was accompanied by modulation of immune homeostasis and inflammatory markers in the Low‐PRS group, whereas such changes were absent in the High‐PRS group (Figure 4).

In the Low‐PRS group, associations between changes in telomere length and immune markers were evaluated. As shown in Table 2, changes in telomere length were significantly associated with several immune markers. In particular, telomere‐length change was inversely associated with changes in GDF15 (*β* = −0.00089, *p* = 0.0289) and IL‐1β (*β* = −0.00095, *p* = 0.0089).

**TABLE 2:** Multivariate linear regression analysis of changes in telomere length in relation to alterations in inflammatory and stress‐related markers.

Phenotypic variable	*N*	*B* (95% CI)	*p*
ΔGDF15 (ng/mL)	61	−0.00089 (−0.0017, −0.00010)	**0.0289**
ΔIgG (ng/mL)	61	−0.00095 (−0.00165, −0.00025)	**0.0089**
ΔIL‐1β (ng/mL)	61	0.018 (−0.076, 0.11)	0.706
ΔTNF‐α (ng/mL)	61	−0.00053 (−0.0011, 0.00005)	0.073

*Note:* *p* values were obtained from multivariable linear regression analyzes conducted in the Low‐PRS group, adjusting for sex and age. Bolded entries fall below the significance threshold of *p* < 0.05.

Abbreviations: IL‐1β, interleukin‐1 beta; TNF‐α, tumor necrosis factor alpha; GDF‐15, growth differentiation factor 15; IgG, immunoglobulin.

### 3.5 Metabolomic Alterations Associated With NTs Supplementation in the High‐PRS and Low‐PRS Groups

The present study suggests that the anti‐aging efficacy of exogenous nucleotides (NTs) varies according to genetic background defined by the urate polygenic risk score (UA‐PRS). NTs supplementation significantly reduced DNA methylation age (DNAmAge) in participants with a high genetic risk, whereas it was mainly related to attenuated telomere shortening and improved immune–inflammatory profiles among those with a low genetic risk. These findings provide preliminary evidence for a potential gene–nutrient interaction in the modulation of biological aging, suggesting that the anti‐aging effects of NTs are shaped by inter‐individual differences in nucleotide metabolism and urate‐associated genetic architecture.

**FIGURE 5:**
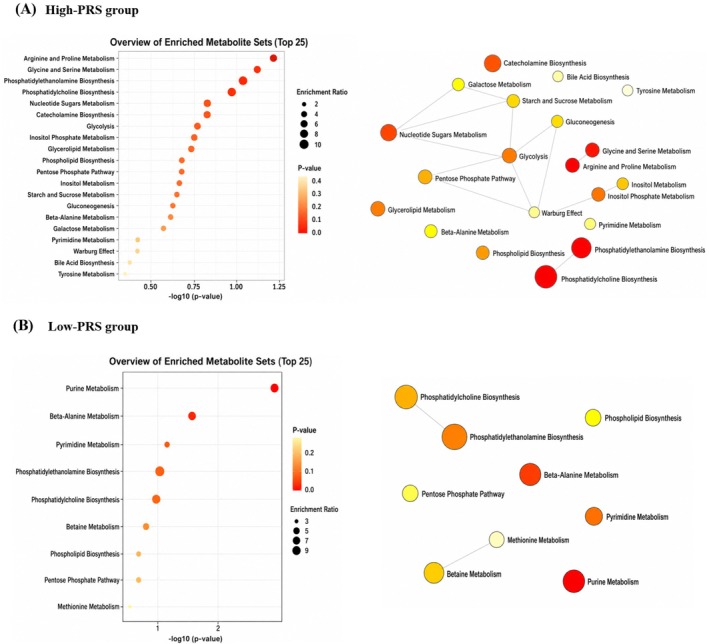
Metabolic pathway enrichment analysis of different UA‐PRS risk groups following NTs intervention. (A) Top 25 enriched pathways in the High‐PRS group (*n* = 60). (B) Top 25 enriched pathways in the Low‐PRS group (*n* = 61).

In the High‐PRS group, several metabolites showed nominally significant alterations following NTs intervention (Table S9). The three most significantly changed metabolites were ureidopropionic acid (*β* = 1.07, 95% CI 0.38–1.75, *p* = 0.0022), 2‐pyrocatechuic acid (*β* = 5.74, 95% CI 1.63–9.85, *p* = 0.0062), and 4‐acetamidobutanoic acid (*β* = −65.37, 95% CI −112.59 to −18.16, *p* = 0.0067). Exploratory pathway analysis suggested potential involvement of glycolysis, starch and sucrose metabolism, pentose phosphate pathway, and nucleotide sugar metabolism. In the pathway network, glycolysis and pentose phosphate pathway appeared to occupy central positions, linking energy and biosynthetic processes (Figure 5A).

In the Low‐PRS group, several metabolites also showed nominally significant alterations after NTs supplementation (Table S10). The three most significantly changed metabolites were uracil (*β* = 29.23, 95% CI 12.92 to 45.53, *p* = 0.00044), guanosine (*β* = −178.08, 95% CI −313.01 to −43.16, *p* = 0.0097), and 2‐phenylglycine (*β* = −137.34, 95% CI −245.59 to −29.08, *p* = 0.013). Exploratory pathway analysis indicated that these metabolites were mainly related to phosphatidylcholine biosynthesis, phosphatidylethanolamine biosynthesis, beta‐alanine metabolism, and purine metabolism (Figure 5B).

## 4 Discussion

The anti‐aging effects of exogenous nucleotides (NTs) may be modulated by genetic background defined by the urate polygenic risk score (UA‐PRS). NTs supplementation significantly reduced DNA methylation age (DNAmAge) in participants with high genetic risk, whereas it primarily preserved telomere length and improved immune–inflammatory homeostasis among those with low genetic risk. These findings indicate that NTs exert distinct metabolic and cellular effects depending on individual nucleotide metabolic capacity, providing support for a genotype‐dependent model of nutritional aging intervention.

The divergence in NTs effects across UA‐PRS groups may arise from differences in purine metabolism and redox background. Urate, the end product of purine degradation, functions as a major extracellular antioxidant but can also promote oxidative stress when produced in excess (Xu et al. 2025; Albuja‐Quintana et al. 2025). Individuals with lower UA‐PRS have reduced urate synthesis and weaker antioxidant buffering, making them more vulnerable to oxidative damage; in these individuals, exogenous NTs may replenish nucleotide pools to support DNA repair and antioxidant defense (Tan et al. 2021). In contrast, high‐PRS individuals have greater purine turnover and oxidative flux (Du et al. 2024), so additional NTs may be redirected into glycolysis‐ and one‐carbon–related pathways to sustain methylation and redox homeostasis (Bernasocchi and Mostoslavsky 2024). This genotype‐dependent redistribution may provide a mechanistic explanation for the distinct anti‐aging effects observed between groups.

In individuals with a low UA‐PRS, exogenous nucleotide supplementation primarily influenced immune–inflammatory and oxidative stress–related pathways. Transcriptomic enrichment revealed upregulation of genes involved in mesenchymal stem cell proliferation, T‐cell differentiation, and immune regulation, alongside downregulation of pathways related to interleukin‐1 signaling and epigenetic suppression. These findings suggest that NTs supplementation modulated transcriptional programs linked to immune balance and stress adaptation. Consistent with these transcriptomic signatures, clinical analyzes showed increased proportions of CD4^+^ T cells and a higher CD4^+^/CD8^+^ ratio, together with decreased levels of IgG, GDF15, and IL‐1β.

Circulating GDF15 has been identified as a stress‐responsive cytokine and a proxy for biological aging, with elevated levels correlating positively with chronological and epigenetic aging indices (Torrens‐Mas et al. 2024). Likewise, IL‐1β is a central pro‐inflammatory cytokine whose chronic elevation is linked to age‐related diseases, disability, and mortality in older adults (Tylutka et al. 2024). The reductions in GDF15 and IL‐1β observed in this study therefore may reflect attenuation of systemic inflammatory and cellular stress signaling. Moreover, changes in telomere length were inversely associated with both markers, consistent with emerging evidence that immune cell composition and inflammatory activity are causally related to telomere length regulation (Liu et al. 2023). Collectively, these results suggest that in low‐PRS individuals, NTs may exert anti‐aging effects primarily through reinforcement of antioxidant defense and immune homeostasis, thereby maintaining genomic stability under conditions of reduced endogenous urate‐mediated antioxidant capacity.

In contrast, participants with a high UA‐PRS showed a distinct transcriptional and metabolic response profile. Transcriptomic analysis identified five differentially expressed transcript features corresponding to four genes, including downregulated SEC14L1, PPP4R2 and ORC5, and upregulated GPBP1. These genes are involved in intracellular lipid transport, phosphatase‐related regulation, cellular stress responses and DNA replication (TeSlaa et al. 2023). Together with metabolomic signals related to glycolytic and PPP‐derived fluxes, these findings suggest that NTs supplementation may help maintain metabolic and epigenetic homeostasis in genetically high‐risk individuals (van Gerwen et al. 2023). However, given the limited number of differentially expressed features, this interpretation should be considered exploratory.

Taken together, these findings suggest that exogenous nucleotide supplementation may exert anti‐aging effects through distinct biological routes associated with genetic background. In individuals with lower urate‐related genetic risk, NTs may primarily enhance immune resilience and reduce inflammatory and oxidative stress, thereby preserving telomere integrity and delaying cellular senescence. Conversely, in high‐PRS individuals, NTs appear to influence metabolic and transcriptional networks centered on glycolytic and pentose phosphate pathways, improving redox balance and epigenetic maintenance. This dual regulatory pattern underscores the metabolic plasticity of nucleotide metabolism and highlights the importance of considering purine metabolic capacity when designing precision nutritional interventions targeting aging.

Several limitations warrant mention. First, the study sample size was moderate and confined to older Chinese adults, limiting the generalizability of findings to other ethnicities. Second, the UA‐PRS explained only part of the variance in serum urate levels; integrating other genetic, epigenetic, and metabolic factors may yield more comprehensive risk stratification. Third, the 19‐week intervention period may not fully capture long‐term effects on biological aging trajectories. Finally, the median split of UA‐PRS resulted in relatively small subgroups, which may have reduced the statistical power for subgroup‐specific multi‐omics analyzes. Therefore, these findings should be interpreted cautiously. Future research should explore longitudinal, multi‐omics analyzes integrating methylome, transcriptome, and metabolome data to elucidate dynamic NTs–gene–environment interactions.

Collectively, this study provides preliminary but novel evidence for a genotype‐dependent model of nucleotide‐based anti‐aging nutrition, offering exploratory mechanistic insights and a foundation for personalized interventions aimed at promoting healthy longevity.

## Author Contributions

Ruisheng Fu conceived and designed the study, performed data analysis, and drafted the manuscript. Shuyue Wang contributed to data interpretation, figure preparation, and manuscript revision. Yuxiao Wu assisted in statistical modeling and pathway enrichment analyzes. Xueying Qin participated in data preprocessing, quality control, and visualization. Tao Huang and Yong Li provided methodological guidance and contributed to the critical review of the manuscript. Meihong Xu supervised the entire project, provided conceptual input, and secured funding. All authors read and approved the final version of the manuscript.

## Funding

The authors have nothing to report.

## Conflicts of Interest

The authors declare no conflicts of interest.

## Supporting information


**Table S1:** Overview of study assessments.
**Table S2:** Associations between UA‐PRS and baseline serum urate.
**Table S3:** Multivariable linear regression analysis of the interaction between NTs intervention and UA‐PRS on changes in Median DNAmAge.
**Table S4:** Multivariable linear regression analysis of the interaction between NTs intervention and UA‐PRS on changes in T/S ratio.
**Table S5:** Characteristics of the participants in the High‐PRS and Low‐PRS group.
**Table S6:** Generalized Estimating Equations of outcomes include baseline and 19‐week.
**Table S7:** Differentially expressed genes in the High‐PRS group (|log2FoldChange| > 1 and adjusted *p*‐value < 0.05).
**Table S8:** Differentially expressed genes in the Low‐PRS group (|log2FoldChange| > 1 and adjusted *p*‐value < 0.05).
**Table S9:** Generalized estimating equations of outcomes include baseline and 19‐week in the High‐PRS group.
**Table S10:** Generalized estimating equations of outcomes include baseline and 19‐week in the Low‐PRS group.

## Data Availability

The data described in the manuscript, including individual‐level genomic and phenotypic data, as well as the code book and analytic code, will not be made publicly available due to privacy and confidentiality concerns. These data contain sensitive personal information, and restrictions apply to their availability to protect participant anonymity.
