# Supplementary material for: Urate‐Related Genetic Risk Modulates the Anti‐Aging Effects of Exogenous Nucleotides: Multi‐Omics Evidence From Older Adults
Source: Aging Cell. 2026 Jun 9;25(6):e70581. doi: 10.1111/acel.70581 (PMC13249583; doi:10.1111/acel.70581)
Supplement: Supplementary file 1 — Table S1: Overview of study assessments. Table S2: Associations between UA‐PRS and baseline serum urate. Table S3: Multivariable linear regression analysis of the interaction between NTs intervention and UA‐PRS on changes in Median DNAmAge. Table S4: Multivariable linear regression analysis of the interaction between NTs intervention and UA‐PRS on changes in T/S ratio. Table S5: Characteristics of the participants in the High‐PRS and Low‐PRS group. Table S6: Generalized Estimating Equations of outcomes include baseline and 19‐week. Table S7: Differentially expressed genes in the High‐PRS group (|log2FoldChange| > 1 and adjusted p‐value < 0.05). Table S8: Differentially expressed genes in the Low‐PRS group (|log2FoldChange| > 1 and adjusted p‐value < 0.05). Table S9: Generalized estimating equations of outcomes include baseline and 19‐week in the High‐PRS group. Table S10: Generalized estimating equations of outcomes include baseline and 19‐week in the Low‐PRS group. [file ACEL-25-e70581-s001.docx]

# Supplementary Contents

[Supplementary Table 1. Overview of Study Assessments 2](#_Toc212036133)

[Supplementary Table 2. Associations between UA-PRS and baseline serum urate 3](#_Toc212036134)

[Supplementary Table 3. Multivariable linear regression analysis of the interaction between NTs intervention and UA-PRS on changes in Median DNAmAge 4](#_Toc212036135)

[Supplementary Table 4. Multivariable linear regression analysis of the interaction between NTs intervention and UA-PRS on changes in T/S ratio 5](#_Toc212036136)

[Supplementary Table 5. Characteristics of the participants in the High-PRS and Low-PRS group 6](#_Toc212036137)

[Supplementary Table 6. Generalized Estimating Equations of outcomes include baseline and 19-week. 8](#_Toc212036138)

[Supplementary Table 7. Differentially expressed genes in the High-PRS group (|log2FoldChange| > 1 and adjusted p-value < 0.05) 9](#_Toc212036139)

[Supplementary Table 8. Differentially expressed genes in the Low-PRS group (|log2FoldChange| > 1 and adjusted *p*-value < 0.05) 10](#_Toc212036140)

[Supplementary Table 9. Generalized Estimating Equations of outcomes include baseline and 19-week in the High-PRS group 12](#_Toc212036141)

[Supplementary Table 10. Generalized Estimating Equations of outcomes include baseline and 19-week in the Low-PRS group 14](#_Toc212036142)

## Supplementary Table 1. Overview of Study Assessments

| **Category** | **Outcome** (**Units**) | **Source/Method** |
| --- | --- | --- |
| **Genetic risk score** | SNPs | Venous blood draw (fasted) |
| **Dietary Assessment** | Accurate food and nutrient intake (g /day) | Photo-Assisted three-day 24-hour Dietary Intake Assessment |
| **Serum urate Measurement** | Serum urate (μmol/L) | Venous blood draw (fasted) |
| **T lymphocyte subset** | CD4+/CD8+ ratio | Venous blood draw (fasted) |
|  | CD3+CD4+ (%) | Venous blood draw (fasted) |
|  | CD3+CD8+ (%) | Venous blood draw (fasted) |
|  | CD3+ (%) | Venous blood draw (fasted) |
| **Inflammatory and**  **humoral immune marker** | IL-1β (pg/ml) | Venous blood draw (fasted) |
|  | TNF-α (pg/ml) | Venous blood draw (fasted) |
|  | GDF-15 (pg/ml) | Venous blood draw (fasted) |
|  | IgG (pg/ml) | Venous blood draw (fasted) |
| **Omics profiling** | Metabolome | Venous blood draw (fasted) |
|  | Transcriptome | Venous blood draw (fasted) |

* SNPs, single nucleotide polymorphisms; CD, cluster of differentiation; IL-1β, interleukin-1 beta; TNF-α, tumor necrosis factor alpha; GDF-15, growth differentiation factor 15; IgG, immunoglobulin.

## Supplementary Table 2. Associations between UA-PRS and baseline serum urate

| **Variables** | **B (95%CI)** | ***p*** |
| --- | --- | --- |
| UA-PRS | 53.68 (21.88, 85.47) | **0.00111** |
| Sex | -77.17322 (-97.93, -56.42) | **2.78×10^-11^** |
| Age | -0.14244 (-3.99, 3.70) | 0.94166 |
| dietary nucleotide intake (g) | 3.36 (-2.17, 8.90) | 0.23139 |

* UA-PRS, the fasting blood glucose polygenic risk score.

* Models were adjusted for sex, age, and dietary nucleotide intake.

* Bolded entries fall below the significance threshold of *p* < 0.05.

## Supplementary Table 3. Multivariable linear regression analysis of the interaction between NTs intervention and UA-PRS on changes in Median DNAmAge

| **Dependent variable** | **Variables** | **B (95%CI)** | **p** |
| --- | --- | --- | --- |
| **Median DNAmAge**  **(years)** | intervention | -1.98 (-4.23, 0.27) | 0.0844 |
|  | UA-PRS | 4.87 (0.37, 9.37) | **0.0343** |
|  | intervention*UA-PRS | -9.03 (-15.99, -2.08) | **0.0114** |
|  | sex | -0.56 (-2.78, 1.66) | 0.617 |
|  | age | -0.05 (-0.47, 0.37) | 0.812 |
|  | dietary nucleotide intake (g) | -0.13 (-0.65, 0.38) | 0.609 |

* Intervention is a binary variable, with 1 indicating the exogenous nucleotides intervention group and 0 indicating the control group.

* UA-PRS, the serum urate polygenic risk score.

* Models were adjusted for sex, age, and dietary nucleotide intake.

* Bolded entries fall below the significance threshold of *p* < 0.05.

## Supplementary Table 4. Multivariable linear regression analysis of the interaction between NTs intervention and UA-PRS on changes in T/S ratio

| **Dependent variable** | **Variables** | **B (95%CI)** | **p** |
| --- | --- | --- | --- |
| **Leukocyte**  **Telomere length**  **(T/S ratio)** | intervention | 0.21 (0.01, 0.4) | **0.0368** |
|  | UA-PRS | 0.48 (0.09, 0.87) | **0.0162** |
|  | intervention*UA-PRS | -0.68 (-1.28, -0.08) | **0.0271** |
|  | sex | 0.09 (-0.1, 0.28) | 0.338 |
|  | age | 0.03 (-0.01, 0.07) | 0.103 |
|  | dietary nucleotide intake (g) | 0.02 (-0.02, 0.07) | 0.358 |

* Intervention is a binary variable, with 1 indicating the exogenous nucleotides intervention group and 0 indicating the control group.

* UA-PRS, the serum urate polygenic risk score.

* Models were adjusted for sex, age, and dietary nucleotide intake.

* Bolded entries fall below the significance threshold of *p* < 0.05.

## Supplementary Table 5. Characteristics of the participants in the High-PRS and Low-PRS group

| **Variable** | **Time** | **High-PRS** | | | | | **Low-PRS** | | | | |
| --- | --- | --- | --- | --- | --- | --- | --- | --- | --- | --- | --- |
|  |  | **NTs group** | | **CON group** | | ***p*** | **NTs group** | | **CON group** | | ***p*** |
|  |  | **N** | **Mean±SD** | **N** | **Mean±SD** |  | **N** | **Mean±SD** | **N** | **Mean±SD** |  |
| Median DNAmAge  (years) | T0 | 34 | 59.88 ± 4.00 | 26 | 56.56 ± 5.60 | **0.014** | 25 | 59.81 ± 4.50 | 36 | 57.77 ± 4.83 | 0.097 |
|  | T1 | 21 | 59.52 ± 7.31 | 18 | 59.38 ± 7.42 | 0.96 | 20 | 59.42 ± 6.33 | 24 | 58.05 ± 4.99 | 0.44 |
|  | T2 | 33 | 53.95 ± 5.84 | 26 | 55.92 ± 4.86 | 0.16 | 24 | 55.59 ± 6.52 | 35 | 54.56 ± 4.79 | 0.51 |
|  | T1-T0 | 21 | -1.40 ± 5.14 | 18 | 4.16 ± 6.81 | **0.0078** | 20 | -0.64 ± 5.46 | 24 | -0.85 ± 5.42 | 0.9 |
|  | T2-T0 | 33 | -6.11 ± 6.07 | 26 | -0.63 ± 4.11 | **0.00013** | 24 | -4.27 ± 5.26 | 35 | -3.39 ± 5.88 | 0.55 |
|  | T2-T1 | 21 | -6.33 ± 5.99 | 18 | -4.74 ± 6.43 | 0.43 | 20 | -3.24 ± 6.76 | 24 | -2.79 ± 4.56 | 0.8 |
| Leukocyte Telomere length  (T/S ratio) | T0 | 34 | 3.28 ± 0.45 | 26 | 3.13 ± 0.53 | 0.23 | 25 | 3.19 ± 0.46 | 36 | 3.37 ± 0.55 | 0.17 |
|  | T1 | 32 | 3.04 ± 0.54 | 25 | 3.06 ± 0.50 | 0.87 | 25 | 3.12 ± 0.42 | 36 | 2.97 ± 0.37 | 0.16 |
|  | T2 | 33 | 2.95 ± 0.42 | 26 | 2.89 ± 0.37 | 0.52 | 24 | 3.00 ± 0.27 | 35 | 2.88 ± 0.34 | 0.14 |
|  | T1-T0 | 32 | -0.26 ± 0.57 | 25 | -0.08 ± 0.48 | 0.21 | 25 | -0.07 ± 0.48 | 36 | -0.40 ± 0.59 | **0.019** |
|  | T2-T0 | 33 | -0.34 ± 0.43 | 26 | -0.24 ± 0.53 | 0.43 | 24 | -0.16 ± 0.33 | 35 | -0.48 ± 0.55 | **0.0074** |
|  | T2-T1 | 31 | -0.07 ± 0.25 | 25 | -0.17 ± 0.34 | 0.25 | 24 | -0.09 ± 0.29 | 35 | -0.07 ± 0.19 | 0.77 |

* **T0** refers to the baseline measurement, **T2** represents the endpoint measurement. **T1-T0** represents the change from **T0** to **T1***,* **T2-T0** represents the change from **T0** to **T2** and **T2-T1** represents the change from **T1** to **T2**.

* The ***p-values*** are derived from *t*-tests that compare the changes between exogenous nucleotides group and Control group.

* Bolded entries fall below the significance threshold of *p* < 0.05.

## Supplementary Table 6. Generalized Estimating Equations of outcomes include baseline and 19-week.

| **Variable** | **Time** | **Mean difference in change between group (NTs group** **vs Control group)** | | | | | |
| --- | --- | --- | --- | --- | --- | --- | --- |
|  |  | **High-PRS** | | | **Low-PRS** | | |
|  |  | **β** | **95%CI** | ***p*** | **β** | **95%CI** | ***p*** |
| Median DNAmAge (years) | T2vsT0 | -5.10 | (-7.71, -2.48) | **0.00013** | -0.82 | (-3.83, 2.19) | 0.59 |
| Leukocyte Telomere length (T/S ratio) | T2vsT0 | -0.08 | (-0.33, 0.17) | 0.53 | 0.31 | (0.10, 0.53) | **0.0043** |

* T0 refers to the baseline measurement, T2 indicates the endpoint measurement, T2vsT0 represents the change from T0 to T2.

* *P* values are based on repeated measures analysis using generalized estimating equations (GEE), with models including the interaction between group assignment and time. Covariates adjusted in the models were baseline values of the outcomes, age, sex, and dietary nucleotide intake.

* Primary assessment timepoints include baseline and 19-week.

* Bolded entries fall below the significance threshold of *p* < 0.05.

## Supplementary Table 7. Differentially expressed genes in the High-PRS group (|log2FoldChange| > 1 and adjusted p-value < 0.05)

| **Variable** | **Gene** | **Log2**  **Fold**  **Change** | ***p*** | ***p_adj_*** | **Expression Trend**  **(NTs vs Control)** |
| --- | --- | --- | --- | --- | --- |
| NM_001204410 | SEC14L1 | -1.10 | 1.39×10^-10^ | 2.67×10^-6^ | Down-regulated |
| NM_001318028 | PPP4R2 | -1.38 | 5.55×10^-10^ | 5.35×10^-6^ | Down-regulated |
| NM_001144001 | SEC14L1 | -1.10 | 2.05×10^-8^ | 4.94×10^-5^ | Down-regulated |
| NM_001127236 | GPBP1 | 1.57 | 5.21×10^-5^ | 0.012413 | Up-regulated |
| NM_181747 | ORC5 | -1.19 | 2.48×10^-4^ | 0.032094 | Down-regulated |

* Differential gene expression analysis was performed using DESeq2.

* *P* values were calculated based on Wald tests within the DESeq2 model. Adjusted *p* values were corrected for multiple testing using the Benjamini–Hochberg false discovery rate (FDR) procedure.

## Supplementary Table 8. Differentially expressed genes in the Low-PRS group (|log2FoldChange| > 1 and adjusted *p*-value < 0.05)

| **Variable** | **Gene** | **Log2**  **Fold**  **Change** | ***p*** | ***p_adj_*** | **Expression Trend**  **(NTs vs Control)** |
| --- | --- | --- | --- | --- | --- |
| NM_001345968 | ZNF394 | -1.14 | 2.08×10^-7^ | 1.11×10^-4^ | Down-regulated |
| NM_001366058 | OTUD4 | -1.77 | 6.10×10^-7^ | 2.14×10^-4^ | Down-regulated |
| NM_001349437 | APOBEC3G | -1.17 | 1.45×10^-6^ | 3.72×10^-4^ | Down-regulated |
| NM_001005526 | SF3B1 | -2.18 | 1.68×10^-5^ | 1.91×10^-3^ | Down-regulated |
| NM_001198978 | SMAP2 | 1.01 | 1.79×10^-5^ | 1.99×10^-3^ | Up-regulated |
| NM_001321250 | VOPP1 | -1.09 | 2.44×10^-5^ | 2.49×10^-3^ | Down-regulated |
| NM_001164098 | VCAN | -1.63 | 1.47×10^-4^ | 7.76×10^-3^ | Down-regulated |
| NM_001320597 | DDX5 | -1.04 | 2.39×10^-4^ | 1.04×10^-2^ | Down-regulated |
| NM_001270841 | CRIP2 | -1.00 | 3.22×10^-4^ | 1.26×10^-2^ | Down-regulated |
| NM_001321592 | CAMK2D | -1.00 | 5.07×10^-4^ | 1.67×10^-2^ | Down-regulated |
| NM_001282117 | RFX3 | 1.03 | 5.47×10^-4^ | 1.75×10^-2^ | Up-regulated |
| NM_001042500 | DEFA1B | 1.13 | 8.85×10^-4^ | 2.37×10^-2^ | Up-regulated |
| NM_001369497 | TBC1D10C | -1.23 | 9.23×10^-4^ | 2.44×10^-2^ | Down-regulated |
| NM_001378077 | GPR137 | -1.59 | 9.62×10^-4^ | 2.49×10^-2^ | Down-regulated |
| NM_001359194 | HLA-DRB1 | 1.64 | 1.27×10^-3^ | 2.91×10^-2^ | Up-regulated |
| NM_002483 | CEACAM6 | 1.19 | 2.11×10^-3^ | 4.00×10^-2^ | Up-regulated |
| NM_023068 | SIGLEC1 | 1.15 | 2.32×10^-3^ | 4.23×10^-2^ | Up-regulated |
| NM_021070 | LTBP3 | 1.31 | 2.32×10^-3^ | 4.23×10^-2^ | Up-regulated |
| NM_001286842 | PHF19 | -1.05 | 2.79×10^-3^ | 4.73×10^-2^ | Down-regulated |

* Differential gene expression analysis was performed using DESeq2, which applies a negative binomial distribution framework as described by Love et al.

* *P* values were calculated based on Wald tests within the DESeq2 model. Adjusted *p* values were corrected for multiple testing using the Benjamini–Hochberg false discovery rate (FDR) procedure.

## Supplementary Table 9. Generalized Estimating Equations of outcomes include baseline and 19-week in the High-PRS group

| **Variable** | **Time** | **Expression Trend (NTs vs Control)** | **Mean difference in change between group**  **(NTs group** **vs Control group)** | | |
| --- | --- | --- | --- | --- | --- |
|  |  |  | **β** | **95%CI** | ***p*** |
| Ureidopropionic acid | T2vsT0 | Up-regulated | 1.07 | (0.38, 1.75) | 0.0022 |
| 2-Pyrocatechuic acid | T2vsT0 | Up-regulated | 5.74 | (1.63, 9.85) | 0.0062 |
| 4-Acetamidobutanoic acid | T2vsT0 | Down-regulated | -65.37 | (-112.59, -18.16) | 0.0067 |
| Phenylglyoxylic acid | T2vsT0 | Up-regulated | 11.77 | (2.59, 20.95) | 0.012 |
| 4-(Methylthio)benzoic acid | T2vsT0 | Up-regulated | 2.87 | (0.53, 5.20) | 0.016 |
| Ethanolamine | T2vsT0 | Down-regulated | -932.60 | (-1723.99, -141.20) | 0.021 |
| Phosphocreatine | T2vsT0 | Down-regulated | -543.03 | (-1027.39, -58.66) | 0.028 |
| Creatine | T2vsT0 | Down-regulated | -582.74 | (-1106.62, -58.86) | 0.029 |
| DOPA | T2vsT0 | Down-regulated | -48.23 | (-93.47, -2.99) | 0.037 |
| Glyceric acid | T2vsT0 | Down-regulated | -27323.40 | (-53241.08, -1405.72) | 0.039 |
| Glucose 6-phosphate | T2vsT0 | Down-regulated | -13.56 | (-26.58, -0.54) | 0.041 |
| Orsellinic acid | T2vsT0 | Up-regulated | 0.66 | (0.02, 1.31) | 0.043 |
| Taurochenodesoxycholic acid | T2vsT0 | Down-regulated | -9493.94 | (-18852.45, -135.42) | 0.047 |

* T0 refers to the baseline measurement, T2 indicates the endpoint measurement, T2vsT0 represents the change from T0 to T2.

* *P* values are based on repeated measures analysis using generalized estimating equations (GEE), with models including the interaction between group assignment and time. Covariates adjusted in the models were baseline values of the outcomes, age, sex, and dietary nucleotide intake.

* Primary assessment timepoints include baseline and 19-week.

## Supplementary Table 10. Generalized Estimating Equations of outcomes include baseline and 19-week in the Low-PRS group

| **Variable** | **Time** | **Expression Trend (NTs vs Control)** | **Mean difference in change between group**  **(NTs group** **vs Control group)** | | |
| --- | --- | --- | --- | --- | --- |
|  |  |  | **β** | **95%CI** | ***p*** |
| Uracil | T2vsT0 | Up-regulated | 29.23 | (12.92, 45.53) | 0.00044 |
| Guanosine | T2vsT0 | Down-regulated | -178.08 | (-313.01, -43.16) | 0.0097 |
| 2-Phenylglycine | T2vsT0 | Down-regulated | -137.34 | (-245.59, -29.08) | 0.013 |
| D-Ribose | T2vsT0 | Down-regulated | -3490.02 | (-6265.34, -714.70) | 0.014 |
| Inosine | T2vsT0 | Down-regulated | -1373.95 | (-2483.62, -264.29) | 0.015 |
| Cyclic AMP | T2vsT0 | Down-regulated | -5.25 | (-9.51, -0.98) | 0.016 |
| Choline | T2vsT0 | Up-regulated | 226.25 | (11.26, 441.24) | 0.039 |
| Ureidopropionic acid | T2vsT0 | Up-regulated | 1.12 | (0.06, 2.19) | 0.039 |
| p-Hydroxyhippuric acid | T2vsT0 | Down-regulated | -439.64 | (-860.69, -18.59) | 0.041 |
| Guanine | T2vsT0 | Down-regulated | -3.35 | (-6.57, -0.12) | 0.042 |
| 7-Ketocholesterol | T2vsT0 | Down-regulated | -32.90 | (-64.78, -1.02) | 0.043 |
| Quinic acid | T2vsT0 | Down-regulated | -5.49 | (-10.90, -0.07) | 0.047 |
| Trimethylamine | T2vsT0 | Up-regulated | 3308.79 | (39.86, 6577.73) | 0.047 |
| 4-Aminohippuric acid | T2vsT0 | Up-regulated | 24.86 | (0.18, 49.55) | 0.048 |

* T0 refers to the baseline measurement, T2 indicates the endpoint measurement, T2vsT0 represents the change from T0 to T2.

* *P* values are based on repeated measures analysis using generalized estimating equations (GEE), with models including the interaction between group assignment and time. Covariates adjusted in the models were baseline values of the outcomes, age, sex, and dietary nucleotide intake.

* Primary assessment timepoints include baseline and 19-week.
